# GraP: platform for functional genomics analysis of *Gossypium raimondii*

**DOI:** 10.1093/database/bav047

**Published:** 2015-05-16

**Authors:** Liwei Zhang, Jinyan Guo, Qi You, Xin Yi, Yi Ling, Wenying Xu, Jinping Hua, Zhen Su

**Affiliations:** ^1^State Key Laboratory of Plant Physiology and Biochemistry, College of Biological Sciences, China Agricultural University, Beijing 100193, China and ^2^College of Agriculture and Biotechnology, China Agricultural University, Beijing 100193, China

## Abstract

Cotton (*Gossypium* spp*.*) is one of the most important natural fiber and oil crops worldwide. Improvement of fiber yield and quality under changing environments attract much attention from cotton researchers; however, a functional analysis platform integrating omics data is still missing. The success of cotton genome sequencing and large amount of available transcriptome data allows the opportunity to establish a comprehensive analysis platform for integrating these data and related information. A comprehensive database, Platform of Functional Genomics Analysis in *Gossypium raimondii* (GraP), was constructed to provide multi-dimensional analysis, integration and visualization tools. GraP includes updated functional annotation, gene family classifications, protein–protein interaction networks, co-expression networks and microRNA–target pairs. Moreover, gene set enrichment analysis and *cis*-element significance analysis tools are also provided for gene batch analysis of high-throughput data sets. Based on these effective services, GraP may offer further information for subsequent studies of functional genes and in-depth analysis of high-throughput data. GraP is publically accessible at http://structuralbiology.cau.edu.cn/GraP/, with all data available for downloading.

## Introduction

As the most significant natural fiber and oil crop in the world, cotton (*Gossypium* spp*.*) is also an industrial raw material and military supply, and is widely used in daily life. The *Gossypium* genus comprises ∼50 species (diploid and polyploid), and is ideal material to study polyploidy and genome evolution. Improving fiber yield and quality under various kinds of abiotic and biotic stresses attracts most of the attention in cotton breeding research.

Some fiber developmental genes have been identified, such as *E6* (1), *GhTub1* (2), *GhSusA1* (3) and *GA20ox* (4). Many genes controlling response to changing environments have been identified in model plants, and only a few stress-related genes have been reported in cotton, such as *GhNHX1* (5), *GhDREB1* (6), *GhNAC1–GhNAC13* (7, 8), *GhMKK1* (9), *GhSnRK2* (10), *GhCIPK6* (11), *GhWRKY40* (12) and *GbRLK* (13). It was reported that overexpressing of *G. hirsutum* sucrose non-fermenting 1-related protein kinase 2 (*GhSnRK2*), which acts as a positive regulator in stress responses, exhibited increased tolerance to drought, cold, abscisic acid (ABA) and salt stresses (10). In addition, Wang *et al*. (12) found that *GhWRKY40* was a multiple stress-responsive cotton WRKY gene and played an important role in regulating wounding- and pathogen-induced responses, its overexpression down-regulated most defense-related genes. With the success of the cotton whole-genome sequencing (14, 15), research on functional genomics has become a major challenge for the scientific community. The widespread use of microarrays and next-generation sequencing is an epitome of high-throughput techniques, which produce massive amounts of omics data on cotton and offer biologists new ideas for cotton functional genomics research. Accordingly, integrating the genomic and transcriptomic data in an online database and mining from the integrated data is essential to maximize utility of these valuable data, and to give cotton researchers further understanding of the complex cellular networks.

Several cotton online databases are currently available, such as CottonDB (16), CMD (Cotton Marker Database) (17), TropGene-DB (18), Cotton expressed sequence tag (EST) database (19) and CottonGen (20). CottonDB contains genomic, genetic and taxonomic information for cotton. CMD provides publicly available cotton simple sequence repeat markers. Furthermore, the Cotton EST database is a platform for cotton EST-related information. CottonGen is an integration and update of publicly available cotton data from CottonDB, CMD and TropGene-DB. However, the databases mentioned above emphasize genomic, genetic, taxonomic and marker data, providing limited information on key functional genes and the relationships between them. These databases are also limited in their utilizing of high-throughput data sets.

Driven by this need, GraP (Platform of Functional genomics analysis in *Gossypium raimondii*) was developed to provide an integration, multi-dimensional analysis and visualization platform for cotton functional genomics research. Up to 2 December 2014, GraP includes: (i) integrated information of two versions of genome sequences and microarray and mRNA-seq data; (ii) different analysis tools, including gene searching and genome browsing, metabolism analysis, protein–protein interaction (PPI) prediction, co-expression network and genome synteny analysis of relative species and (iii) other user-friendly interfaces such as GSEA (gene set enrichment analysis) (21) and *cis*-element significance analysis tools with downloadable results. With these integrated information and web tools, GraP will further broaden the omics data access and improve the accuracy and robustness of cotton functional genomics analysis. We also hope it will provide some inspiration for cotton researchers, and further speed up research on cotton yield and quality.

### Data sources

The genome assembly sequences and gene structural annotations of *G. raimondii*, which was the first completed sequence of cotton species, used in GraP are the JGI version from Phytozome (15, 22) and the BGI version from CottonGen (20), respectively (Supplementary Table S1). To get an updated annotation of predicted genes from these assemblies, we used *Arabidopsis* TAIR10 version (23) and closely related species to re-annotate the genome using BLAST (basic local alignment and search tool). The functional annotations of proteins were downloaded or performed respectively from MAPMAN (24), Kyoto Encyclopedia of Genes and Genomes database (KEGG) (25) and InterProScan (26). Furthermore, motif information was downloaded from the plant *cis*-acting regulatory DNA elements (PLACE) database (27). We also collected microRNA data from the miRBase (28) and published literature (29). The cotton microarray probes were downloaded from the Affymetrix official website (http://www.affymetrix.com/estore/). Microarray and RNA-seq data were respectively collected from Gene Expression Omnibus (GEO, http://www.ncbi.nlm.nih.gov/geo/) in the National Center for Biotechnology Information (NCBI) (Supplementary Table S2).

## Materials and Methods

### Gene family classifications

**Figure 1.**
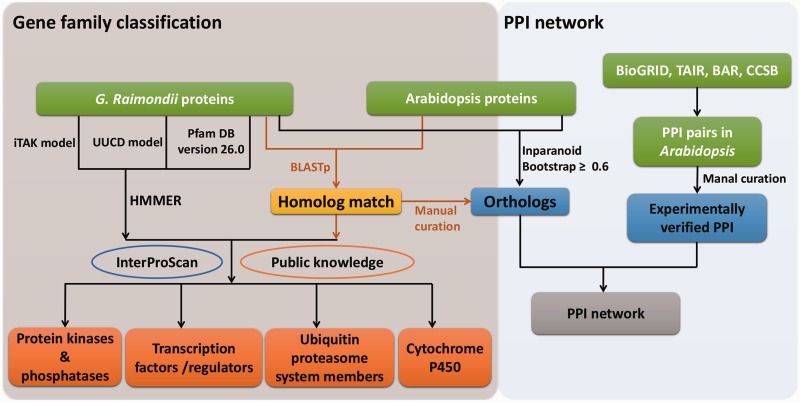
Workflow of gene family classifications and PPI network. HMM models from UUCD, iTAK and Pfam databases were used to search putative members of TFs/TRs, PKs/PPs, UPS and CYP450s. Homolog searches between *G. raimondii* and *Arabidopsis*, and InterProScan as well as public information were also applied to further curate the results. Experimentally assayed PPIs in *Arabidopsis* were retrieved from publicly available data bases, and a giant PPI network was generated by combining orthologs between *G. raimondii* and *Arabidopsis* identified by different methods.

Because transcription factors/regulators (TFs/TRs), protein kinases/phosphatases (PKs/PPs), ubiquitin proteasome system (UPS) members and CYP450s are functionally important in signaling pathways, related gene family classifications were carried out by the strategies summarized in Figure 1. Initially, Hidden Markov Model (HMM) models and well-trained parameters from UUCD (30) and unpublished iTAK databases (http://bioinfo.bti.cornell.edu/cgi-bin/itak/index.cgi) were used respectively to predict putative UPSs, TFs/TRs and PKs/PPs. Additionally, HMMER 3.0 (31) was used to determine proteins that contained conserved domains, annotated with ‘protein phosphatases’, ‘histidine kinases’ and ‘tyrosine phosphatase’*.* Meanwhile, proteins containing the ‘PF00067’ domain were considered as CYP450s according to the Pfam (http://pfam.xfam.org/) annotation.

After that, homologs between *G. raimondii* and *Arabidopsis* were identified by bidirectional BLASTp (e-value ≤ 1e^−^^5^). Information from public databases, such as CYPSI (32), plantsUPS (33), the Cytochrome P450 Homepage (http://drnelson.uthsc.edu/CytochromeP450.html), AGRIS (34) and plantTFDB (35) was further used to consolidate the primary results and filter out ambiguities. Only the member was retained when its family classification was accordant with that of their corresponding *Arabidopsis* homologs in at least one public database. Meanwhile, conserved functional domains searched by InterProScan (26) were adopted to curate the classification results (Table 1). Taking TFs/TRs as an example, 6629 proteins belonged to this category according to the results of a HMM search against the iTAK model. Then, we used information from AGRIS and plantTFDB to manually screen these putative TFs/TRs, and 223 proteins without supporting information from the two public databases were discarded.

**Table 1.** A list of data integrated in GraP

Category	Description	Details
Genome assembly	JGI version	1033 chromosome/scaffolds, 37 505 genes and 77 267 transcripts/proteins
Gene family	TFs/TRs	82 TFs/TRs families, 3275 genes, 6406 proteins
	PKs/PPs	96 PKs/PPs families, 1956 genes, 4292 proteins
	CYP450s	42 CYP450 families, 373 genes, 490 proteins
	UPS	21 UPS member, 1749 genes, 3943 proteins
Functional annotations	Gene ontology	1338 accessions (496 for biological progress, 156 for cell component and 687 for molecular function), 20 979 genes, 45 743 proteins
	KEGG pathways	2815 pathways, 5580 genes, 9532 proteins
	MAPMAN	2662 accessions, 24452 genes, 52 689 proteins
Conserved domain	InterProScan searches	2815 IPR ID, 30343 genes, 64 913 proteins
Intracellular network	Protein–protein interactions	12 483 nodes and 103743 edges
	Gene co-expressions	20 480 nodes, 1 419 237 positively correlated edges and 1 127 237 negatively correlated edges
microRNAs	Mature microRNAs and their targets	416 Precursors with 416 mature sequences, 7506 target mRNAs
Homology match	*A. thaliana*	Inparanoid: 15 142 ortholog gene pairsLevel 1: 18 696 *Arabidopsis* genes match with 21 390 genesLevel 2: 11 679 *Arabidopsis* genes match with 18 144 genes17 167 *Arabidopsis* genes have 72 655 homolog protein matches
	*Theobroma cacao*	16 973 genes in *T. cacao* have 66 568 homolog protein matches
	*Populus trichocarpa*	16 625 genes in *P. trichocarpa* have 63 146 homolog protein matches
	*Ricinus communis*	13 834 genes in *R. communis* have 60 900 homolog matches
Synteny	Blocks	14 344 co-linear blocks covering 37 223 genes

In addition, family classifications for these categories in GraP were manually curated with the following two steps: (a) conserved functional-domain based method: we used InterProScan to identify the domains for all proteins, and then selected the candidates for specific gene families based on conserved functional domains; (b) homology-based method: For those identified candidates derived from step (a), we checked if their best homologs in other relative species (such as *Arabidopsis*) belong to the corresponding gene families or not. Especially, for some ambiguous proteins which have certain domains but with low scores, we further manually aligned these domains to their homologs to avoid some false positives. After that, the remained members were finally released. In the meanwhile, classifications and manual curations for other gene families are undergoing and the results will be released in near future.

### MicroRNAs and targets

MicroRNAs play important roles in transcriptional regulation through impacting the stability and fate of their binding target mRNAs (36). More and more functionally important microRNAs have been identified in various conditions, such as salt, cold and drought environments, with the help of high-throughput technology (e.g. microarray, miRNA-seq and sRNA-seq). Thus we collected 296 microRNAs from miRBase (28) and 127 microRNAs from the recently published literature (29). Because some of them were not identified under the genomic background of JGI version, the genomic locations of their precursors were relocated by the BLAT tool (37). In addition, RNAfold (38) was applied to obtain the pre-miRNA secondary structures and the positions of mature microRNA. We further removed the microRNAs with redundant genomic positions, and finally integrated 416 microRNAs from those resources (shown in Supplementary Table S3). In order to establish the relationships between microRNAs and mRNAs, the psRNATarget web service (39), TargetFinder (40, 41) and TAPIR (42) were employed to find their downstream binding mRNA targets.

### PPI prediction

Intracellular signals are mainly transmitted through PPIs (43). However, only a few PPIs have been experimentally identified, and so it is essential to perform high-quality prediction of PPIs, which will benefit understanding of regulatory relationships between genes. In total, 18 014 experimentally assayed PPIs in *Arabidopsis* were collected from BioGRID (44), TAIR (23), BAR (45) and CCSB (46).

We determined 15 142 ortholog pairs between *Arabidopsis* and *G. raimondii* using Inparanoid (Table 1), using a cutoff of bootstrap ≥0.60 (47). Meanwhile, orthologs were selected from homolog match results by bidirectional BLASTp between *Arabidopsis* and *G. raimondii* (e-value ≤1e^–5^). The top three hits from cotton-to-*Arabidopsis* BLASTp results were inspected and further divided into two levels: (i) level 1 if these hits occurred in the top three in the *Arabidopsis*-to-cotton BLASTp output and (ii) level 2 if these hits occurred in the top 10 (Table 1). Then, 29 399 cotton genes were mapped to 19 169 genes.

With these ortholog matches, PPIs were further predicted according to the collected experimentally verified PPIs in *Arabidopsis*. Since hubs containing closely connected nodes were usually functionally related to each other, the network was divided into 93 closely connected hubs (each hub with at least 10 nodes) using MCODE (fluff = 0.9) (48), which is a tool to find hubs by evaluating the topological structure of a network.

### Gene co-expression network

Due to the lack of transcriptional profile data sets for *G. raimondii* and the high similarities between *Gossypium* subspecies, cotton microarray data sets related to different developmental stages and stresses (Supplementary Table S2) were collected from GEO to construct the cotton gene co-expression network. Microarray elements were also downloaded from the Affymetrix official website (http://www.affymetrix.com/estore/), and used to search for gene–probe matches.

First, the probe consensus sequences were aligned to transcripts by BLASTn with e-value ≤1e^−^^3^ and cumulative identity percentage of consensus ≥60, and 22 524 probes matched to 39 963 transcripts. Second, all CEL files were preprocessed by gcRMA (Guanine Cytosine Robust Multi-Array Analysis) algorithm (49). For each pair of probe sets i and j, all microarray expression values were used to calculate PCC (Pearson’s correlation coefficient) values. Then, the probe-set pairs were retained as expression-correlated if their corresponding PCC ≤ –0.7 or ≥0.7. The expression patterns of two probe sets are similar or positively correlated if their corresponding PCC ≥ 0.7 and vice versa.

### Cis-element significance analysis

There were 394 different motif factors scanned in the upstream of 37 223 genes using the PLACE web service (27). Users can submit their gene sets to GraP to scan for motifs. In order to facilitate users to perform significance analysis of these motifs, the *z*-score of each scanned motif and its corresponding *P*-value are computed with the formulas that follow (50):

z−score=Ni−meaniσiP-value=1−pnorm(Ni,meani,σi)

Where *N*_i_ represents the number of occurrences of motif i in the submitted gene batch, and mean_i_ and *σ*_i_ are the mean and standard deviation of the number of detected occurrence of motif i in 1000 randomly selected genes, respectively. Except for the analysis of known genes, sequences of fasta format can also be submitted for *de novo* motif recognitions.

### High-throughput data integrations

Although genome-wide PPIs and co-expression networks are well constructed, understanding gene expression profiles in tissues under different environmental conditions will greatly increase researchers’ knowledge of gene functions. Since ambiguity still existed for intracellular expression levels of interested genes of not-good-enough matches between cotton microarray and *G. raimondii*, 16 available RNA-seq data sets of *G. raimondii* collected from GEO (Supplementary Table S2) were analysed. The quality of reads were evaluated using FASTX-Toolkit and reads of high quality (at least 80% of bases per read have a score of ≥20) were retained. Then TopHat was utilized to align these reads to the reference genome permitting four mismatches considering their germplasm variances. FPKMs (Fragments Per Kilobase of transcript per Million mapped reads) of transcripts or genes were calculated by cufflinks employing default parameters.

## Results

**Figure 2.**
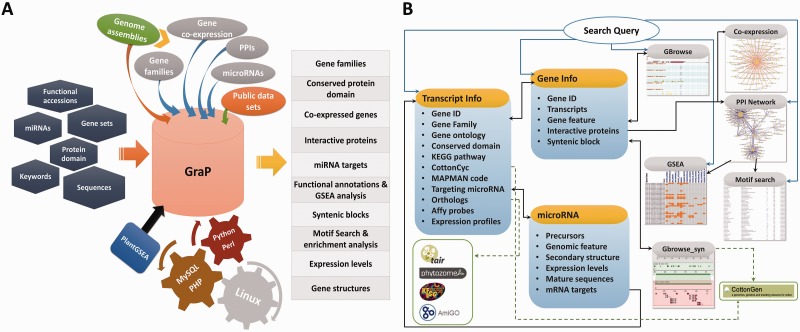
An overview of GraP. (**A**) GraP was developed under LAMP (Linux, Apache, MySQL and Python/Perl) environments and based on integrating analysed results and tools. Several types of queries can be submitted for retrieving relevant information. (**B**) All data sets are organized by offering several web pages, search tools and web services. Meanwhile, extra links with other related public databases are also supplied.

All the processed and analysed results were well integrated. This included gene families comprising 6406 TFs/TRs, 2284 UPS members, 490 CYP450s and 4292 PKs/PPs, 103743 PPIs between 12483 proteins (Supplementary Figure S1), the gene co-expression network containing 1 419 237 positively and 1 127 237 negatively correlated pairs between 20 480 probe sets, and 416 microRNAs as well as their corresponding precursors (Table 1). PHP + MySQL + Javascript + Python/Perl were used to develop application platforms facilitating cotton research, and Cytoscape Web (51) was employed to supply users with a retrieval device to search for PPIs or co-expressed genes. Furthermore, BLAST and GBrowse (52) as well as other widgets were developed for users to freely and directly find the integrated information related to their genes or proteins of interest, comprising all integrated knowledge mentioned above and other fundamental functional annotations (GO, KEGG and MapMan), conserved protein domain, homologs or orthologs of relative species and gene expression levels in different tissues at various time points (Figure 2A).

**Figure 3.**
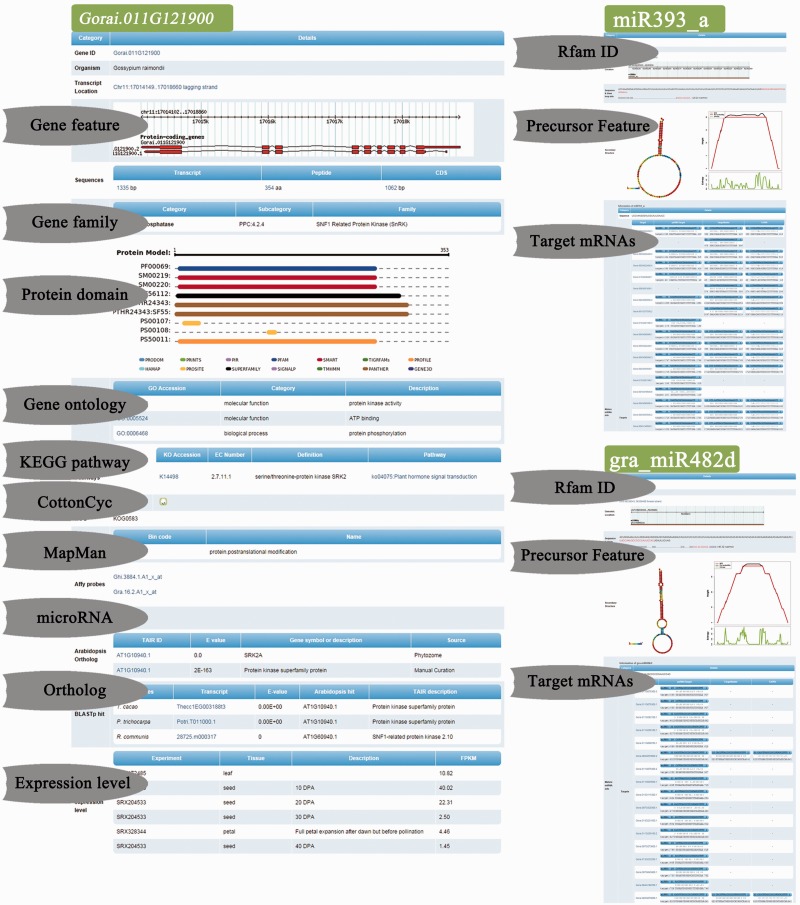
Functional details of *Gorai.011G121900*, miR393 and miR482 in GraP. This page shows the functional details of *Gorai.011G121900* including gene family, targeting microRNAs, conserved protein domains, ortholog matches with other species and expression levels in different tissues.

**Figure 4.**
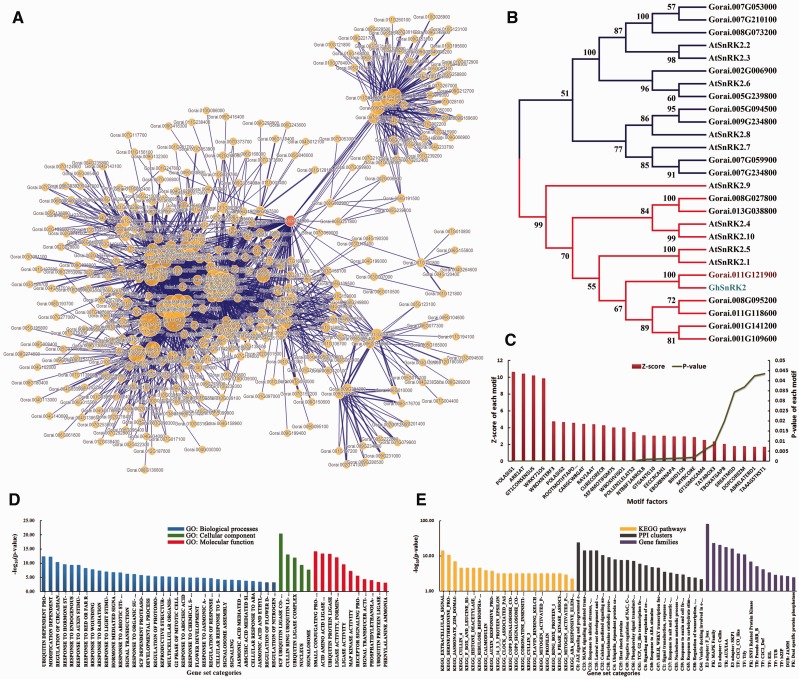
Functional analysis of protein-encoding genes related to *Gorai.011G121900*. (**A**) A PPI network containing *Gorai.011G121900* and its connected signaling key elements. (**B**) A neighbor-joining (NJ) tree of SnRK proteins was performed based on the kinase domains using CLUSTALW and MEGA4 with bootstrap 1000 replicates, only the clades with bootstrap value higher than 50 were shown. (**C**) *Cis*-element enrichment analysis of all interactors showed high significance of signal transduction and key transcription factors. (**D**) GSEA analysis showed that major functions focused on response to abiotic stresses and regulation of related biological processes. (**E**) Enrichment analysis of KEGG pathways, gene families and PPI clusters.

With the help of GraP, users can directly search genes in GBrowse (52), gene structures and their expression levels are displayed and more details (as mentioned above) are also available (Figure 2B and 3). Furthermore, annotation items or keywords can also be submitted to find specific information through the search tools provided in GraP. Apart from this fundamental information in GraP, every gene is hyperlinked to CottonCyc pathways integrated in CottonGen (Figure 2B). In addition to this static information, dynamic clues including PPIs and co-expressed probe sets (Figures 2B and 4A) can further benefit understanding of the biological processes that a gene is involved in. This would benefit key gene selections and relevant experimental designs.

Compared with single gene verification, high-throughput technologies provide large amounts of omics data and give biologists more related information, usually related to specific phenotypes or agronomic traits. Subsequent statistical analysis of functions of the detected differentially expressed (RNA-seq or microarray) or modification enriched (TF binding, histone modification or DNA methylation) genes is routinely carried out to illustrate the related biological phenomena. Therefore, GraP offers users GSEA and *cis*-element significance analysis tools for relevant analysis of their gene batches. Taking PPI hubs as an example, GSEA analyses on them are mainly related to various signaling-mediated or stress-responsive physiological processes, including cell reproduction, defense response, ubiquitin-mediated protein degradation and regulation of gene expression (Supplementary Table S4).

To make query results more believable, homologs in relative species are mapped to cotton genes using BLAST and also linked to other public databases for more information (Figure 2B). Because there were already two different genomic assemblies of *G. raimondii* (14, 15), syntenic blocks between them were scanned with SyMAP (53). Meanwhile, transcripts in each pair matched with BLASTn were retained if both of them belonged to the same block. GBrowse_syn was used to visualize these results in GraP (Supplementary Table S5) (54), which will help cotton biologists to take full advantage of two genome assemblies. Additionally, users can carry out personal searching or update requests through e-mail or leaving messages in GraP, and this will undoubtedly accelerate GraP improvement.

## Discussion

There is no doubt that a novel and comprehensive method of integration and analysis for cotton omics data is essential to maximize utility of publicly available data, and will help to accelerate cotton research. However, a powerful mining tool for omics data has not previously been available. Differing from CottonGen, CottonDB or CMD, GraP is believed to fill the vacant fields for functional analysis. GraP is more suitable for molecular mechanism-driven investigations of gene batches derived from high-throughput sequencing or microarrays, especially for signal transductions under stresses, and will offer researchers some insights into their studies. GraP provides comprehensive knowledge about a specific gene, including family categories, potential interactive partners, *cis*-elements, microRNAs and their downstream targets, as well as possible regulatory pathways.

Taking *SnRKs* as an example, in *G. raimondii*, *Gorai.011G121900* is a member of the SnRK family (Figure 3) and the ortholog of *GhSnRK2* (Figure 4B). It was shown that in the PPI network (Figure 4A), *Gorai.011G121900* could directly interact with 100 other proteins, including F-box (Gorai.013G113800), CUL1 (*Gorai.008G009700*), EBF1 (*Gorai.009G058000*), bZIP TFs (*Gorai.013G258300* and *Gorai.006G111600*), COI1 (*Gorai.011G279900*) and AREB3 (*Gorai.009G301400*). Most of these PPIs have already been verified in *Arabidopsis* (46, 55) (Supplementary Table S6).

In addition, GSEA analysis of all interactors of *Gorai.011G121900*, such as Tify, WRKY TFs and various E3 ligases, showed significant enrichment in the response to light stimulus and other important hormones, including ABA and jasmonic acid (Fisher exact test, FDR ≤ 0.05) (Figure 4D and Supplementary Table S8). Statistical analysis of motifs in the 3000 bp upstream sequences of these protein-coding genes showed that they were significantly related to stress and hormone signaling as well as partially tissue-specific motif factors, such as WRKY71OS, WBOXNTERF3, RAV1AAT and ABRELATERD1 (*P*-value ≤ 0.05; Figure 4C). This showed that these interactors might be transcribed by WRKY TFs and impacted by ABA or other stress-related key elements. It had also been reported that the overexpression of *GhSnRK2* in *Arabidopsis* led to reduced water loss under salt and drought stresses as well as the up-regulation of other key elements, such as AtABI3, AtCBF1 and AtABI5 (10). Furthermore, GhSnRK2 silencing in cotton plants results in a phenotype of alleviated drought tolerance (10). Gra.16.2.A1_x_at was the corresponding probe set of *Gorai.011G121900* and it had 157 positively co-expressed probe sets. Gene ontology enrichment analysis of these probe sets using agriGO (56) exhibited that response to water and chemical stimuli as well as the regulation of gene expression were significantly enriched (*P*-value ≤ 0.05) (Supplementary Figure S2 and Table S7). Moreover, MIR393 is involved in auxin signaling and controlled leaf development under salt stress, and MIR482 is a family of disease resistance-related miRNAs (57, 58). Both MIR393 and MIR482 participate in the posttranscriptional regulation of transcribed mRNAs from the interactors of *Gorai.011G121900* (Figure 3). In summary, all the mined information illustrated that *Gorai.011G121900* was involved in tissue development, hormone and stress resistance (Supplementary Table S8).

However, information in GraP is mainly constructed *in silico*. False-positives or false-negatives can exist due to parameter and method selections during prediction processes and lack of experimental evidence. Therefore, subsequent manual updates are important for error rectification and knowledge supplementation, guaranteeing a good service for user requests. Users can also leave messages or write e-mails to GraP for personal problems.

## Conclusions

GraP is a user-friendly and up-to-date database and analysis platform for functional genomic studies in *G. raimondii*. At present, we have integrated two versions of *G. raimondii* genome data, microarray and mRNA-seq data in the database (Table 1). We have also developed a series of functional analysis tools such as cotton gene family analysis, PPI prediction, co-expression network web service, GSEA analysis, *cis*-element significance analysis toolbox, genome synteny analysis among relative species and other general tools in the database. We hope this will improve the accuracy of cotton functional genomics analysis, and further deepen understanding of gene regulatory networks for effective crop improvement. GraP is freely available at http://structuralbiology.cau.edu.cn/GraP/, and will be updated every 3–4 months with the development of cotton research, as well as the manual curated gene family available.

## Funding

This research was supported by grants from the Ministry of Science and Technology of China (31171276) and the Ministry of Education of China (NCET-09-0735).

## Supplementary Data

Supplementary data are available at *Database* Online.

*Conflict of interest*. None declared.

Supplementary Data
