# Supplementary material for: GraP: platform for functional genomics analysis of Gossypium raimondii
Source: Database (Oxford). 2015 May 16;2015:bav047. doi: 10.1093/database/bav047 (PMC4433718; doi:10.1093/database/bav047)
Supplement: Supplementary Data [file supp_2015_bav047_index.html]

Supplementary Data 

# GraP: platform for functional genomics analysis of *Gossypium raimondii*

## Supplementary Data

files

- Supplementary Data - zip file
