# Supplementary material for: GraP: platform for functional genomics analysis of Gossypium raimondii
Source: Database (Oxford). 2015 May 16;2015:bav047. doi: 10.1093/database/bav047 (PMC4433718; doi:10.1093/database/bav047)
Supplement: Supplementary Data [file supp_bav047_suppl_data.zip › New Microsoft Office Word Document.docx]

**Supplementary files**

**Supplementary Figure S1.** Distribution of node connections. There are 103743 edges (interactions) between 12483 nodes (proteins) and the majority of nodes have no more than 10 neighbors.

**Supplementary Figure S2.** GO functional enrichment analysis of all co-expressed probe sets of Gra.16.2.A1_x_at. (**A**) 158 co-expressed probe sets of GraAffx.29731.1.S1_a_at, the representative probes of SnRK in cotton microarray. (**B**) REVIGO functional summaries after GO enrichment analysis (FDR ≤ 0.05) in agriGO showed that these probe sets were related to signal transduction and response to water stimulus and other related biological regulations. The legend of bubble color is in upper right-hand corner, and indicates the user-provided log10(p-value); size indicates the frequency of the GO term in the underlying GOA database (bubbles of more general terms are larger); according to the manual of REVIGO, semantic x and y coordinates were derived by applying multidimensional scaling to a matrix of the GO terms' semantic similarities ([56](#_ENREF_56),[59](#_ENREF_59)).

**Supplementary Table 1.** Summary of data sources.

**Supplementary Table 2**. Details of collected high-throughput data sets.

**Supplementary Table 3**. Source of collected microRNAs.

**Supplementary Table 4.** Functional descriptions of clusters in PPI network.

**Supplementary Table 5**. Statistical results of syntenic analysis of the assemblies between JGI and BGI version.

**Supplementary Table 6.** List of genes encoding physically interacting proteins of *Gorai.011G121900*.

**Supplementary Table 7.** GO enrichment analysis of co-expression network of Gra.16.2.A1_x_at.

**Supplementary Table 8.** Details of GSEA analysis of the interactors of *Gorai.011G121900.*
