# Supplementary figures and images for: GraP: platform for functional genomics analysis of Gossypium raimondii
Source: Database (Oxford). 2015 May 16;2015:bav047. doi: 10.1093/database/bav047 (PMC4433718; doi:10.1093/database/bav047)

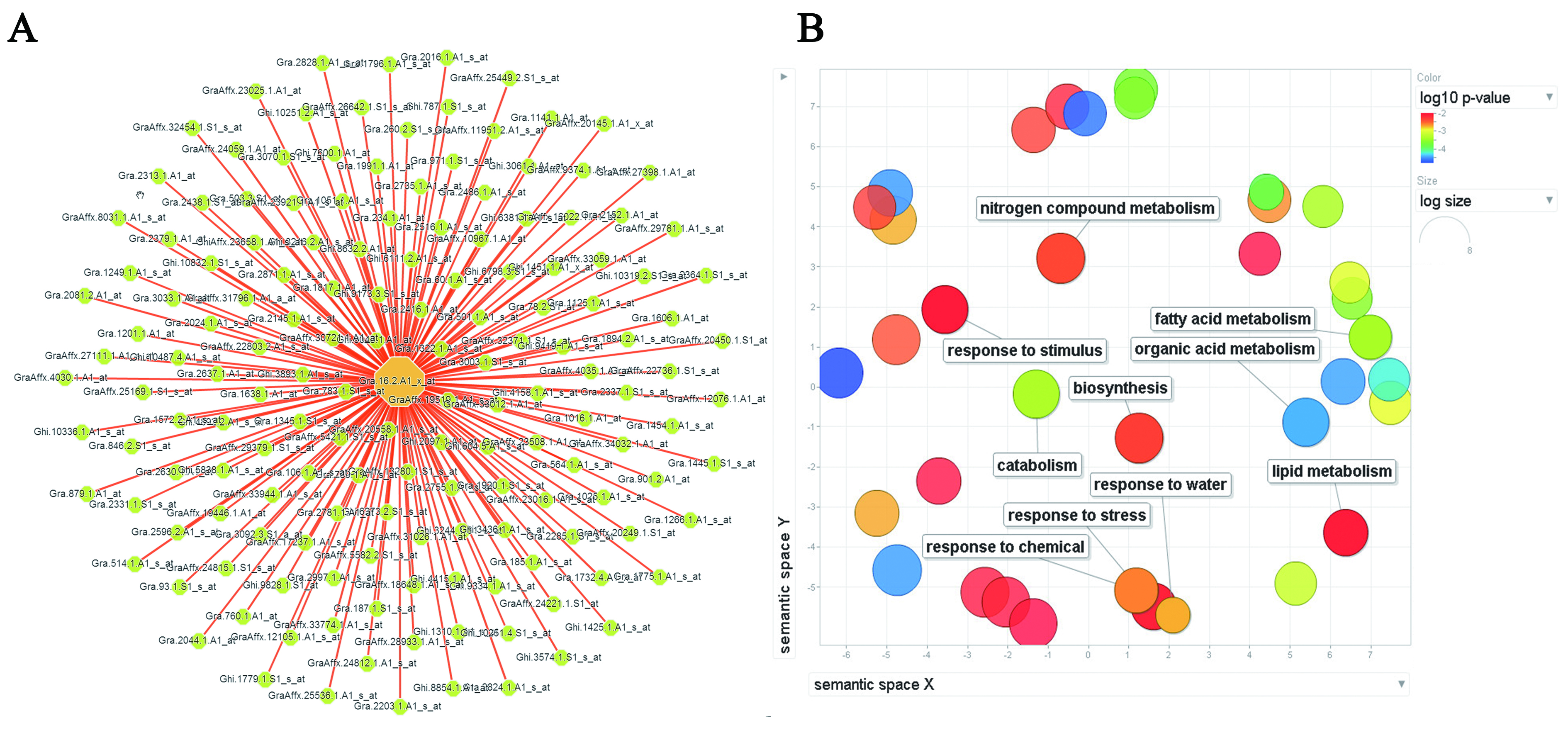

Supplement: Supplementary Data [file supp_bav047_suppl_data.zip › Figure_S2.jpg]

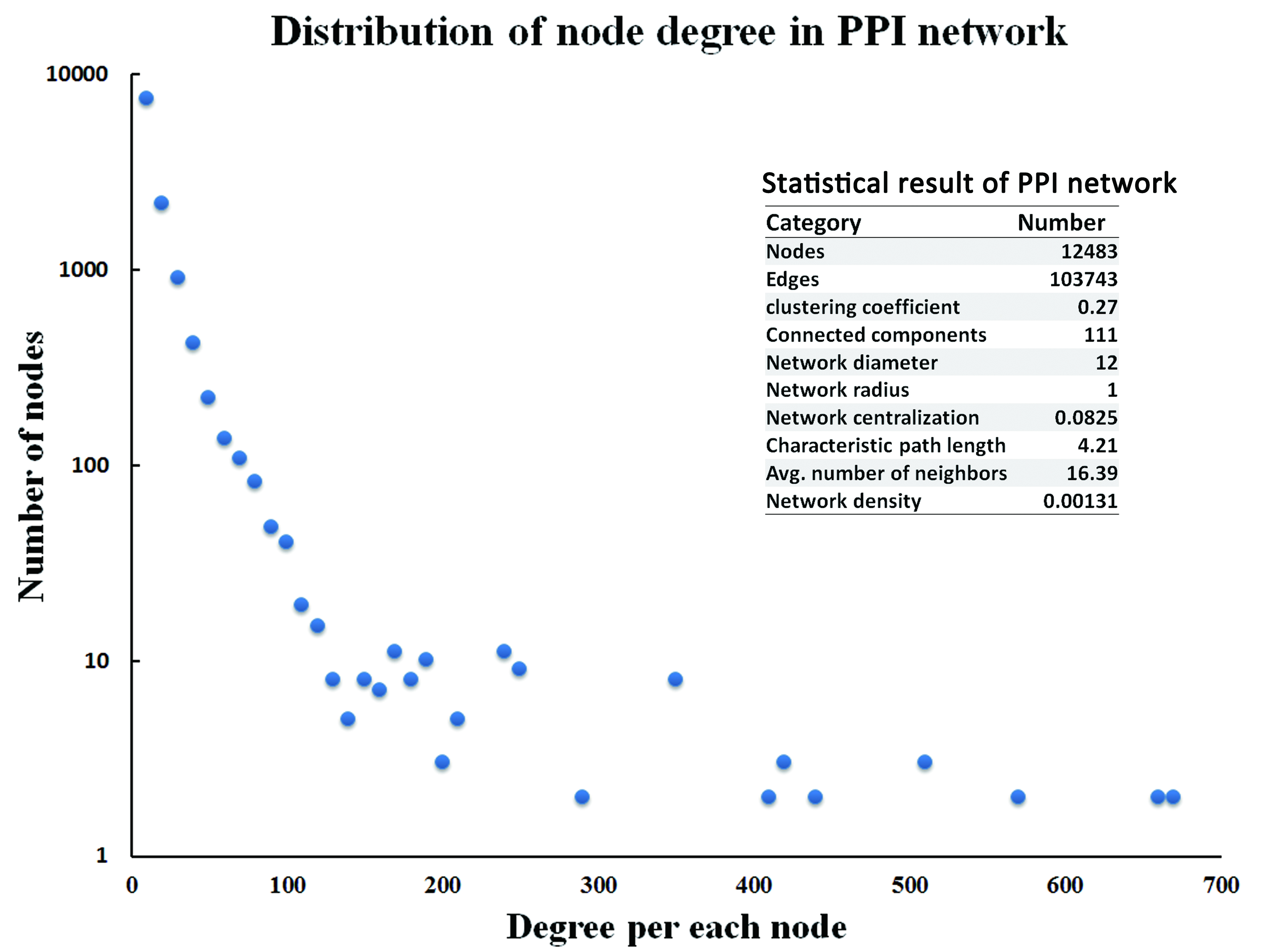

Supplement: Supplementary Data [file supp_bav047_suppl_data.zip › Figure S1.jpg]
